# Oxidative Stress Is a Concept, Not an Indication for Selective Antioxidant Treatment

**DOI:** 10.3390/antiox12061188

**Published:** 2023-05-30

**Authors:** Dov Lichtenberg, Ilya Pinchuk, Eleni Yonassi, Daniela Weber, Tilman Grune

**Affiliations:** 1 Department of Physiology and Pharmacology, Sackler School of Medicine, Tel Aviv University, Tel Aviv 6997801, Israel; 2 Department of Digital Medical Technologies, Holon Institute of Technology, Holon 5810201, Israel; 3 Department Molecular Toxicology, German Institute of Human Nutritio Potsdam-Rehbruecke (DIfE), 14558 Nuthetal, Germany; 4 Food4Future (F4F), c/o Leibniz Institute of Vegetable and Ornamental Crops (IGZ), Theodor-Echtermeyer-Weg 1, 14979 Grossbeeren, Germany; 5 German Center for Diabetes Research (DZD), 85764 Munich-Neuherberg, Germany; 6 German Center for Cardiovascular Research (DZHK), Partner Site Berlin, 13357 Berlin, Germany; 7 Institute of Nutrition, University of Potsdam, 14558 Nutmeal, Germany; 8 Department of Physiological Chemistry, Faculty of Chemistry, University of Vienna, 1090 Vienna, Austria

**Keywords:** quantitation of oxidative stress, biomarkers, antioxidants, selective treatment

## Abstract

The steady-state redox status is physiologically important and therefore homeostatically maintained. Changes in the status result in signaling (eustress) or oxidative damage (distress). Oxidative stress (OS) is a hard-to-quantitate term that can be estimated only based on different biomarkers. Clinical application of OS, particularly for selective antioxidant treatment of people under oxidative stress, requires quantitative evaluation and is limited by the lack of universal biomarkers to describe it. Furthermore, different antioxidants have different effects on the redox state. Hence, as long as we do not have the possibility to determine and quantify OS, therapeutic interventions by the “identify-and-treat” approach cannot be assessed and are, therefore, not likely to be the basis for selective preventive measures against oxidative damage.

## 1. Introduction

In this commentary, we present our view on the relationship between the results of clinical trials and basic research on oxidative stress (OS) and antioxidants. First, we present a brief history of the clinical applications of the term OS. Next, we relate to the major problems associated with the possibility of clinical applications, including the complexity of the qualitative definition of the term OS and the effects of antioxidants [1,2,3,4,5]. Our aim is to evaluate the possibility of future clinical applications based on the concept of OS.

## 2. Brief History of Redox Biology and Medicine

In 1956, Harman raised the free radical hypothesis of aging [6]. Since then, many diseases were attributed to oxidative damage caused by reactive oxygen species as a result of excessive production or too-slow elimination of free radicals. OS is a “global concept in redox biology and medicine” [3,4,5,7]. Unfortunately, the term OS, as defined by Helmut Sies in 1985, is qualitatively defined, with no measurable criterion. The assessment of OS is based on the concentrations of various biomarkers of a different chemical nature, mostly biomarkers of oxidative damage to biomolecules, including malondialdehyde (MDA), protein carbonyls (PC), nitrotyrosine (NT) and oxidized LDL (oxLDL). The problem is that the different methods yield different results. As a consequence, OS depends on the chemistry of the biomarker used to estimate it and the degree of standardization of these measurement methods [8,9].

The evidence for a causal relationship between basic research and clinical applications was rather weak, as noted by Sies in his review titled “Oxidative stress: From basic research to clinical application”. In this review he wrote: “Current evidence in clinical research does not show unequivocal destination between causal or associative relationship of pro-oxidant to the disease process” [1]. Nevertheless, a group of 17 leading scientists in the field of OS and antioxidants signed “the Saas Fee (Switzerland) declaration” in June 1992. Based on their estimate that “antioxidant nutrient may have major significance in the prevention of a number of diseases”, the declaration calls for public support of research and education. In spite of this apparent wide agreement at the time, one has to note that this point of view is largely overcome and outdated.

In 2004, two meta-analyses reported that the indiscriminate consumption of vitamin E results in a reduction in life expectancy [10,11]. Moreover, Rahal et al. raised doubt regarding the effects of healthy foods that reduce OS [12]. Although under debate, these analyses at least put the strategy of the universal use of antioxidants in disease prevention under doubt.

In 2017, Ghezzi et al. reviewed the epidemiological aspect of “The oxidative stress theory of disease” and “discussed the levels of evidence required to claim causality in preclinical research on OS” [13]. Relating the weakness of “the oversimplification associated with the OS theory of disease”, they noted that “no systematic clinical trials demonstrated health promotion, disease prevention and/or positive longevity effects in humans”. However, the authors expressed hope that several recent topics are about to be clinically applied and that the progress in drug development will enable a more effective use of antioxidants. Nevertheless, more and more evidence from basic research shows that endogenous antioxidative defenses and exogenous low-molecular-weight antioxidants (LMWA) are the main contributors to reactive species detoxification. In other words, in spite of disappointments, we still hope that under certain, defined conditions, the targeted use of antioxidants might be helpful.

## 3. Hope

In view of the physiological importance of changes in oxidative status, such changes have been vaguely subdivided into two classes of OS, denoted as “distress”, which causes chemical damage, and “eustress”, providing beneficial signaling. The term “eustress” has to be used with care; signaling by “eustress” can be a positive reaction.

The term “distress” is supposed to mean “chemical damage”, whereas both the terms eustress and distress, similar to the term OS, are not chemical situations, but global concepts that cannot be chemically distinguished, being the same in all cases. Drug therapy is a common approach in preventive medicine. Good examples are treatment strategies for high blood pressure and high cholesterol. In both cases, if the level is too high, the person will have to be appropriately treated. Using this approach, it was advocated to treat “core problems” such as inflammation [14]. The redox steady state is important in preserving the correct functionality of cellular processes [1].

## 4. Identify-and-Treat Approach

Both oxygen and nitrogen active species (ROS/RNS), regardless of whether they were formed via biochemical processes or environmental pollutants (e.g., radiation), damage cellular biomolecules (lipids, carbohydrates, proteins, and nucleotides). Such damage is reflected by “biomarkers of OS” (Supplemental Table S1).

Several defense systems act within cells to prevent an uncontrolled OS increase. The major contributors to our protection against oxidative damages are the antioxidative enzymes, superoxide dismutase (SOD) and catalase, as well as the glutathione system. All can be activated when needed under OS conditions. With the aid of nonenzymatic LMWA present in foods or occurring endogenously, we are well-protected against oxidative damages.

The antioxidative action of LMWA is far from being well understood [14]. The obvious mechanism involves the quenching of up to two free radicals per one molecule of the LMWA. The most popular assays of antioxidant capacity and OS evaluation are based on this mechanism. Interestingly, an antioxidant-derived free radical may also interact with a molecule rather than with another free radical, thus antioxidants can play roles as pro-oxidants, by initiating the oxidation of other molecules. Such a molecule might be another antioxidant, therefore, creating an antioxidative redox chain (e.g., vitamin E and vitamin C).

In redox medicine, the “identify-and-treat” approach assumes that people under high OS are likely to benefit the most from any antioxidant supplementation [15,16,17,18,19,20]. The application of the “identify-and-treat” approach, of course, requires the quantitation of OS and knowledge on the effects of LMWA. Both of these demands are problematic: OS is qualitatively defined and its level depends on how it is evaluated. Moreover, different antioxidants have different effects on the OS, as discussed below.

## 5. Quantitation of Oxidative Stress

The quantitation of OS, as defined by Sies in 1985 [1,2], requires the practically impossible measurement of both the rate of free radical production and the rate of their elimination. In general, the extent of OS largely depends on endogenous concentrations or flux rates of reactive oxygen species (ROS). Accordingly, for any OS-related criterion to be of clinical value, such as hydrogen peroxide or a superoxide anion, we have to relate to the implication of the fact that the level of OS appears to depend on the method used to estimate the OS. Because a universal criterion does not exist, the integration of multiple biomarkers has been considered (Supplemental Table S2), but this is too complex and unprecise to serve as a routine assay. In addition, the prediction of the OS concept that OS should be “cured” by antioxidants is inconsistent with extensive evidence that shows that antioxidant supplementation is rarely used as an antioxidant medication/compound. Notably, many different diseases and pathological states were associated with different OS biomarkers (Table 1). Several natural compounds, mostly herbal, including curcumin, polyphenols and vitamins, are used in traditional/complementary medicine, but it is not clear whether these compounds act as antioxidants or rather via inducing an endogenous response or other mechanisms.

The qualitatively defined concept denoted as OS is an apparently unresolvable problem: data derived from various methods do not correlate (or correlate poorly) with each other (e.g., PC and MDA), which means that OS cannot be defined by a universal criterion. Accordingly, no single factor can describe the whole redox status. We think that this means that there are several “types” of OSs that differ in biomarkers. According to Ghezzi et al., type-“zero” biomarkers indicate the direct measurement of ROS in clinical settings. Type-“one” biomarkers, including the oxidation products of cellular molecules by ROS, are the most frequently used indicators of OS, with these including the oxidation products of lipids (such as MDA, or isoprostanes), proteins (mostly PC) or nucleic acids (particularly 8-hydroxydeoxyguanosine, 8-OH-dG). Type-“three” biomarkers include LMWA and antioxidant enzymes.

**Figure 1:**
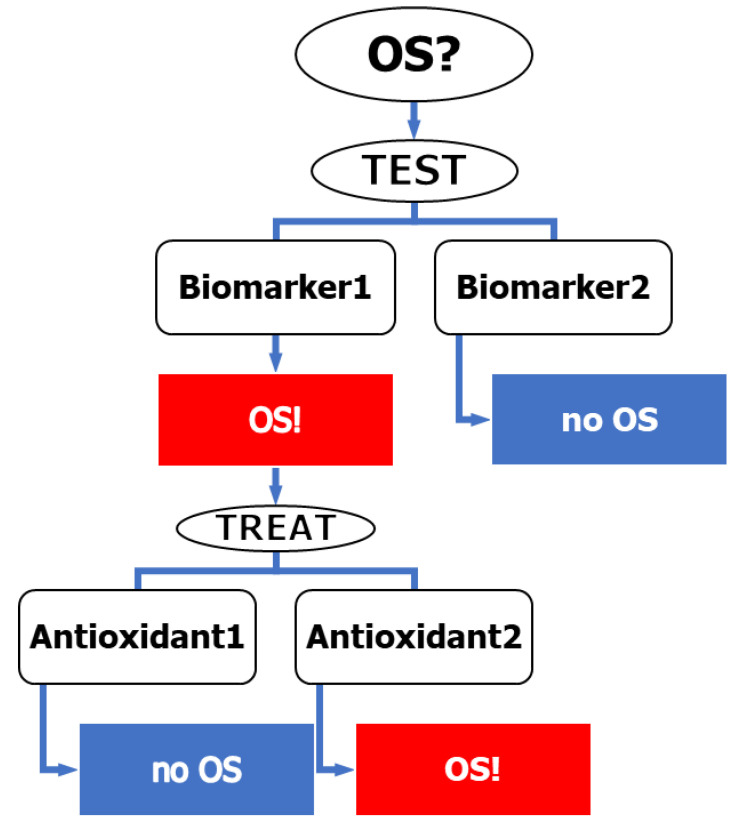
A scheme of the ”identify-and-treat” protocol. Different biomarkers may give different results of the OS level. Treatment of people under OS by different antioxidants may lead to different and even opposite results.

In relating to the possibility of applying the reasonable “identify-and-treat” approach, Witztum suggested that the problems are the lack of reliable measures of determination of OS and evaluating the antioxidative activity of the alleged antioxidants [20]. In fact, the problem is more complex than that not only because of the difficulties in quantitating the qualitatively defined term OS, but also because of the lack of knowledge on the possible treatments of OS. Consequently, even if we knew how to identify people under OS, we could not have general advice on how to treat them (Figure 1).

For the qualitatively defined concept denoted as OS to be used as a risk factor of oxidative damage, it has to relate to the excess of pro-oxidative compounds over antioxidants. In other words, we need to define the OS quantitatively such that it reflects a person’s capability to resist oxidative damage. OS is commonly evaluated by the steady-state concentration of the oxidation products that serve as the biomarkers of OS.

In fact, antioxidant supplementation rarely protects against any disease, maybe because it does not achieve protective levels in vivo (i.e., not high enough to adequately scavenge free radicals). Moreover, LMWAs may have functions unrelated to their antioxidant capacity. In some cases, antioxidant treatment produced unwanted results. Sometimes, the ROS action is the desired effect of the treatment [17]. Given the trend of the increasing consumption in antioxidants, it is important to emphasize that the commonly used approach of identifying OS and treating “people under OS” with antioxidants [18,19,20] might be a misuse of the OS concept, because no single biomarker can be used to evaluate the “general redox state” and different antioxidants (the oxidative effect of tocopherol in LDL is well-documented) [34]. This effect, denoted by tocopherol-mediated peroxidation (TMP), may be quite common because the antioxidative effect of LMWA usually occurs via the bi-radical quenching of antioxidant-derived free radicals [14]; however, under certain conditions, the latter radicals interact with molecules and the resultant free radicals mediate propagation, where some antioxidants may promote the oxidation of specific biomolecules. The relevance of such mechanisms within in vivo processes is still questionable.

Based on the assumption that people under high OS are likely to benefit most from antioxidant supplementation, Witztum (1998) raised an objection against the indiscriminate supplementation of vitamin E having a reliable measure of determination of OS and evaluating the antioxidative activity. This approach, denoted as “identify-and-treat”, is very common in medicine, although it is often described by different words (e.g., test or measure or find). The general use of this commonly recommended approach in redox biology is unfortunately not based on data, because on one hand, no biomarker reflects the general “redox state”, whereas on the other hand, the effects of different LMWA are not the same [14].

In view of its essential physiological roles, the redox steady status is strictly maintained through complex homeostatic mechanisms. Although OS has been associated with a wide spectrum of pathological conditions, in many cases, it is difficult to establish a causal relationship between OS and disease progression. The comprehensive study of the European consortium, MARK–AGE, yielded data on the OS of more than 2000 age-stratified healthy volunteers, as evaluated on the basis of more than 10 different biomarkers [14,35,36]. The analysis of these data revealed that the answers to each of the three important questions depends on the choice of the biomarker. First, the lists of the people with the top 10% OS, according to ten different methods, overlapped not more than expected for a random choice [8].

## 6. Assay Kits

For a kit to be used routinely, it has to be reproducible, sensitive, rapid, and easy to conduct, and if only possible, be highly throughput. Furthermore, it has to be specific to allow for a conclusion based on the result of the test. Today, kits are commercially available from many producers, many of whom produce more than one kit to assay cellular OS and the concentrations of ROS in body fluids, aiming at identifying individuals under OS who are believed to gain from the supplementation of antioxidants. The main lipid peroxidation products are hydroperoxides, aldehydes and isoprostanes. The latter products have been highly recommended, but both hydroperoxides and aldehydes are more commonly used for screening OS. This can be attributed to the need for the pre-treatment of biological fluids.

Hydroperoxides are relatively stable in biological systems, as long as they are not exposed to non-chelated transition metal ions. By contrast, an ex vivo assay of OS, commonly measured in dilute serum in the presence of transition metal ions, occurs mostly via a radical pathway, because the products of lipid peroxidation (hydroperoxides) interact with the transition metal ions, producing new free radicals. The resultant self-accelerated free-radical chain reaction may yield information on the reactions of questionable relevance to peroxidation in vivo, where hydroperoxides are relatively stable.

The term d-ROM is used as abbreviation for determination of reactive oxygen metabolites. The d-ROM estimates of OS are commonly expressed in terms of Carrately units [17,18]. In healthy subjects, the d-ROMs have a value between 250 and 300 Unit CARR (U CARR). Values above 300 U CARR are observed under “condition of oxidative stress”, either as environmental (e.g., cigarette smoking, alcohol abuse, inadequate exercise, diets unbalanced on a quality and quantity level) or diseases associated with changes in oxidative balance (e.g., cardiovascular diseases, neurodegenerative disorders, metabolic syndrome or cancer). In fact, there are claims that ROM can be used to assess disease risk, but this use is limited by uncertainty about the chemical nature of ROM. Specifically, the ROM test is standardized relative to tert-butyl hydroperoxide or H_2_O_2_. Thus, the obtained serum concentrations of hydroperoxides from this assay appear unrealistically high.

The use of MDA concentration to evaluate the in vivo OS appears to be better than the presumably steady-state (homeostatic) concentration of hydroperoxides. First, the concentration of MDA differs markedly from that of hydroperoxides. This may explain the lack of correlations between OS determined by MDA and determined by d-ROM. Second, the total MDA (T-MDA, including bound and free MDA), unlike d-ROM, differed between smokers and non-smokers, which led to conclude that this is the best marker to detect OS. Other research considers F2-isoprostanes the most reliable OS biomarkers (depending on the analytical method and sampling used to determine isoprostanes), and they are therefore the reliable markers of oxidative damage to lipids. However, they cannot always be used as a common marker of OS.

## 7. Concluding Remarks

Several lines of indirect evidence indicate that some specific groups of people may gain from the supplementation of vitamin E or other antioxidants. The possible selective use of antioxidant supplementation is based on the reasonable, but problematic, paradigm that people under OS are those who are likely to benefit the most from treatment. If this is right, it justifies the routine measurements of OS, so long as OS is determined using a method that the evaluation of OS depends an appropriate method.

We think that the reason for the dependence of OS on the method of its evaluation is the existence of several types of OS [7], that the various OS biomarkers might have a different biological fate and that, in turn, antioxidants affect various types of OS differently. We therefore suggest that the classification of various types of OS, as well as of their association with different pathologies and their response to different antioxidants, are important issues. Until such information is available, the level of OS, as determined by any method, is of limited value. The specific groups that may benefit from specific antioxidant supplementation maybe, but are not necessarily, those that suffer from high OS of one type or another. This does not mean that it is not worthwhile assaying OS.

We believe in the opposite conclusion: the classification of OS is very important and the only way we can achieve it is to assay OS by more than one assay.

## 8. Future Perspectives

Thirty years after Sies noted that “clinical research does not show causal relationship of pro-oxidant to the disease process”, “there is still no direct evidence for causality” [1]. Furthermore, the “identify-and-treat” approach could have been expected to enable the monitoring of OS, and thus “improve patient management decision and patient outcomes” and reduce the “overall cost of care” [17,18]. Unfortunately, taken together, the studies described above indicate that the “identify-and-treat” approach is not likely to yield a selective treatment of high OS. There are attempts to develop a method to evaluate the “general oxidative status” that does not depend on biomarkers.

One such possibility is to use the lag preceding rapid peroxidation ex vivo, to assay the resistance of lipoprotein or unfractionated serum lipids to peroxidation [37,38,39,40] and the concentration of an antioxidant that doubles the lag to rank antioxidants [41,42]. Remarkably, this approach also mostly considers the damage to lipids. Hence, the oversimplified “identify-and-treat” approach does not address many important questions, such as the chemistry and source of ROS in different organs and organelles, the stability and distribution of biomarkers, the distribution of antioxidants (lipid/water, tissue, etc.) and the action of the antioxidants via transcription factor activation. Therefore, the measurements of the steady-state concentrations of different biomarkers are of very limited practical value. Moreover, given the “double-edge sword nature” of antioxidants, even if we had a way to identify people under OS, we do not have a straightforward way of treating OS with LMWA.

In view of the large number of oxidants and the differences between the reactivity of different targets, the concentration of a single biomarker cannot be expected to give an answer to the overall capacity of an individual to resist oxidative damage, and can therefore not be of diagnostic value [8]. The latter conclusion is supported by the finding that the overlapping of the lists of people with the top 10% OS, according to different criteria, is not larger than expected for a random choice. We must note that the MARK-AGE participants were generally healthy, and the results will be potentially different in other populations with underlying diseases.

In a recent publication, Sies et al. categorically testified against the possibility of using the total antioxidative capacity (TAC) to assess the OS [2] (a point of view we share). Instead, they proposed assessing the activities of individual antioxidant enzymes, but acknowledged that in view of the very large number of oxidants and the huge differences between the reactivity of different substrates, the concentration of a single biomarker cannot be expected to give an answer to the overall capacity of an individual to resist OS.

Table 1 shows that the biomarker that correlates with the largest number of diseases is MDA. We think that this finding indicates that the products of a reaction of free radicals with molecules were the products of the interaction with PUFA residues. Nevertheless, other biomarkers are likely the products of free-radical oxidation of other substrates. We think that certain diseases are associated with specific biomarkers. The type of OS and the treatment may be identified according to the disease. The latter requires further research. An overview of selected biomarkers of OS and OS indices can be found in the supple-mental material [33,43,44,45,46,47,48,49,50,51,52,53,54,55]. In addition, other relevant biomarkers might still be missing from the “commonly used panel” and the sampling time could also affect the results. For instance, once changes in MDA are detected in a patient, it may be too late to intervene. Perhaps proteomic/mass spectrometry changes, phosphorylation patterns of specific OS-related proteins, or transcriptomic changes of particular genes could provide earlier information in terms of clinical application.

Transcriptomic or proteomic profiles of OS-related proteins could be disease- and organ-specific, and provide clues to the signaling pathways altered under OS. Rather than direct antioxidant therapy, targeted intervention in OS-related pathways using agonists/inhibitors may be effective in controlling the damaging effects of OS. However, these techniques and their output are not yet large-scale, standard clinical practice. This requires more experimentation.

## Figures and Tables

**Table 1:** Pathologies and biomarkers.

Pathology	Biomarker Found	Control (*N*)	Participants (*N*)	Reference
PD	8-OHdG, MDA, nitrite, ferritin	6037	MA. 80 studies, *N* = 7212	[21]
CVD	8-OHdG OxLDL	1106 1727	MA. 14 studies, *N* = 810 prospective population-based case–cohort study, *N* = 333	[22,23]
Cancer, solid tumors	8-OHdG, γH2AX		MA. 21 studies, *N* = 2121	[24,25]
COPD	MDA	530	MA. 14 studies, *N* = 817	[26]
AMD	MDA	656	MA. 12 studies, *N* = 634	[27]
Aging	PCOOH	Young, 20	Aged 20	[28]
Atherosclerosis	oxLDL		MA. 12 studies, *N* = 8644	[29]
DM	AOPPs, MDA	30 30	DM patients 24 75	[30,31,32]
Alzheimer’s disease	HO-1, 8-OHdG, HNE, isoprostanes, PCO, nitrotyrosine, AGEs		Review, 97 published articles	[33]

The main pathologies that are linked to the specific biomarkers of OS based on the citations above and a review recently published about the biomarkers measuring inflammation and OS. (MA. = meta-analysis ; *N* = number of participants). Abbreviations see below.

## Supplementary Materials

The following supporting information can be downloaded at: https://www.mdpi.com/article/10.3390/antiox12061188/s1, Table S1: Selected Biomarkers of Oxidative Stress; Table S2: OS Indices.

## Abbreviations

*General terms*	
GPx	Glutathione peroxidase
LMWA	Low-molecular-weight antioxidants
OS	Oxidative stress
PUFA	polyunsaturated fatty acid
ROS	Reactive oxygen species
SOD	Superoxide dismutase
*Diseases*	
AMD	Age-related macular degeneration
COPD	Chronic obstructive pulmonary disease
CVD	Cardiovascular disease
DM	Diabetes mellitus
PD	Parkinson’s disease
*Indices*	
AOPP	Advanced Oxidation Protein Products
CARR	Carrately
HO-1	Heme oxygenase-1
LMWA	Low-molecular-weight antioxidants
(ox)LDL	(oxidized) Low-density lipoprotein
MDA	Malondialdehyde
NT	Nitrotyrosine
PCOOH	Phosphatidylcholine Hydroperoxide
(d-)ROM	(determination of) Reactive oxygen metabolites
TAC	Total antioxidant capacity
8-OHdG	8-hydroxy-2′-deoxyguanosin
