# Supplementary material for: Oxidative Stress Is a Concept, Not an Indication for Selective Antioxidant Treatment
Source: Antioxidants (Basel). 2023 May 30;12(6):1188. doi: 10.3390/antiox12061188 (PMC10295793; doi:10.3390/antiox12061188)
Supplement: Supplementary file 1 [file antioxidants-12-01188-s001.zip › antioxidants-2389717-supplementary.pdf]

antioxidants

MDPI

Supplemental Table S1. Selected Biomarkers of Oxidative Stress.

	Biomarker	Definition	Reference
**Biomarkers associated with damage to lipids**	**MDA**	Malondialdehyde is frequently used to assess indication of lipid peroxidation caused by free radical damage to lipids of the cellular membrane or lipoproteins.	[43]
	**oxLDL**	Oxidized low-density lipoprotein (OxLDL) is likely to play an important role in atherogenesis by promoting an inflammatory [2]environment and lipid deposition in the arterial wall. It can be measured as one biomarker for oxidative damage of cell lipids.	[44]
	**HNE**	Lipid peroxidation end products may be useful as OS biomarkers, with a study suggesting that 4-hydroxy-2-nonenal protein adducts appear to be particularly interesting to use for *in vivo* evaluation.	[45]
	**PCOOH**	Phosphatidylcholine hydroperoxide has been found to be linked to an excess of oxidants over antioxidants and believed to be a reliable biomarker of oxidative damage.	[46]
**Biomarkers associated with damage to DNA**	**8-oxo-dG**	Widely used as a biomarker for oxidative stress and carcinogenesis because it is a free radical induced oxidative lesion form. This can be identified in nuclear and mitochondrial DNA, 8-hydroxy-2' -deoxyguanosine (8-OHdG) or 8-oxo-7,8-dihydro-2' -deoxyguanosine (8-oxo-dG).	[47]
**Biomarkers associated with damage to proteins**	**PC**	In human cells, serum protein carbonyls increase when OS levels rise due to oxidative damage to proteins.	[48]
	**AOPPs**	Advanced oxidation protein products, formed mainly by chlorinated oxidants resulting from activity of myeloperoxidase. Clinical evidence has supported the valuable potential of AOPPs as biomarkers of oxidative injury. This biomarker has been found to correlate with the development and progression of diseases and chronic conditions related to inflammatory status and immune dysregulation.	[49]
	**Nitrotyrosine**	Nitrotyrosine is a stable modification formed by nitrosative stress. Nitrotyrosine is now thought to be a key biomarker of lipid peroxidation and NO/NO_2 reactions and of OS.	[50]
**Other biomarkers**	**AGEs**	Advanced Glycation Endproducts (proteins or lipids glycated after exposure to sugars) are formed endogenously and are present in many tissues, e.g. also in amyloid plaques in Alzheimer's Disease.	[33]
	**HO-1**	Heme oxygenase-1 is a rate-limiting enzyme catalyzing oxidative degradation of cellular heme to liberate free iron, carbon monoxide (CO) and biliverdin in mammalian cell. Expression of heme-oxygenase-1, is considered an important marker of oxidative stress.	[51]
	**Ferritin**	Due to ferritin's role in the detoxification of iron, it may also serve as a biomarker for OS. Serum ferritin is also a well-known clinical marker.	[52]
	**Nitrate**	Nitric oxide has been identified as the molecule responsible for endothelial dependent vasodilatation. In OS the formation of peroxynitrite from the reaction of NO with the superoxide anion might be important	[53,54]

Supplemental Table S2. OS Indices.

Index	Definition	Methods
Oxidative Balance:	ROS-TAC	Calculated by measuring chemiluminescence. The computation of the ROS-TAC score is complex so this ratio was not used further.
Glutathione Ratio	GSSG/GSH ratio	The ratio is assessed from GSH to GSSG or from GSSG to GSH indicating intracellular OS.
Thiol Ratios	-SH group of thiols/ oxidized form disulphide -SS- ratio	The ratio relates to cysteine and glutathione in their reduced and oxidized forms.
Oxidative Stress Index (OSI)	Total oxidant/antioxidant ratio of a biological sample.	The OSI considers several components of OS measured together, by assessing the total oxidant and antioxidant of a biological sample. Examples include: FRAP,TAOP,ORAC, TAA, TAC, TAS,TRAP, TOC and TOS.
Oxidative INDEX	Measurement of hydroperoxides by dROMs/ serum antioxidant activity ratio	This is similar to the OSI but the oxidative part of the index is derived from is the measurement of total hydroperoxides by dROMs and to the serum antioxidant activity has used a procedure called the OXY Adsorbent Test.<br>Its calculation method is difficult.
OXY Score	Summation of F-MDA, T-MDA, GSSG, GSH, iPF2α as the combined oxidative damage.	This score includes individual measurements of different parameters of the oxidative balance from serum and urine.<br>antioxidant enzymes are not included, limiting the value of the score.
Oxidative Stress Score	Balance between SOD, GPx and CAT	This score takes the main antioxidant enzymes (SOD, GPx and catalase (CAT)) into account.

Different indexes of measuring OS.

Tabulated summary of the main Indexes used to access OS based on a recently published meta-analysis by Sanchez-Rodriguez et al. [55].

Abbreviations

GPx	Glutathione peroxidase
OS	Oxidative stress
ROS	Reactive oxygen species
SOD	Superoxide dismutase
MDA	Malondialdehyde
(d-)ROM	(determination of) Reactive oxygen metabolites
TAA	Total antioxidant activity
TAC	Total antioxidant capacity
TAOP	Total antioxidant potential
TAS	Total antioxidant status
TOC	Total oxidative capacity
TOS	Total oxidative status
TRAP	Total radical-trapping antioxidant parameter
